# Artificial intelligence in medical education: Are we ready for it?

**DOI:** 10.12669/pjms.36.5.3042

**Published:** 2020

**Authors:** Nazish Imran, Masood Jawaid

**Affiliations:** 1 Dr. Nazish Imran, MBBS, FRCPsych, MRCPsych (London), MHPE, Associate Professor, Department of Child & Family Psychiatry, King Edward Medical University/Mayo Hospital, Lahore, Pakistan; 2 Dr. Masood Jawaid, MBBS, MCPS, MRCS (Glags), FCPS (Surg), MHPE, Director Medical Affairs and Pharmacy Services, PharmEvo, Associate Editor, Pakistan Journal of Medical Sciences, Consultant Surgeon, Darul Sehat Hospital, Karachi, Pakistan

“Our fate is to change.” Enrico Coiera emphasized in Lancet, while discussing fate of medicine in the time of Artificial intelligence (AI).^1^ The rapid growth of artificial intelligence in healthcare around the globe are glimmers of a future, where AI driven tools are likely to define the way medicine will be practiced in 21st century. Artificial Intelligence (AI) or the mimicking of human cognition by computers is conceptualized as a machine with intelligent behavior like reasoning, perception, ability to generalize and learn from experience.^2^ Literature suggests that AI systems and tools can help to deliver precision medicine, be faster, effective and as accurate as human clinicians and improve delivery of healthcare.^3^-^6^ Although in their infancy now but with continuation of trend seen so far, Robotic surgery, e-Patients etc. are likely to be standard practice in future medical practice.^7^,^8^ One need to realize that future medical practice will be partnership between physicians and allied healthcare professionals, machines and patients. With AI set to impact on every aspect of healthcare, new roles for health professionals will emerge requiring new medical education.

Medical educationists throughout the world are of the view that a reboot of medical education and curriculum shift from “Knowledge acquisition” to emphasis on “Knowledge management and communication” is needed to address the emerging challenges of 21^st^ century especially the increasing integration of big data and artificial intelligence in professional practice.^9^ Medical students of today will experience all the opportunities and challenges associated with use of AI in medicine throughout their career as future doctors. Current medical education does not prepare future physicians for the impending AI revolution in healthcare. As rightly pointed out that “Educational standards need to be refreshed, refined and improved as technology changes and data fog thickens”.^10^ Apart from understanding of basic sciences and its connection with clinical sciences, medical education curriculum for 21^st^ century needs to include content to improve future physicians’ capacity to practice in data rich environment supported by AI. Medical students need to have solid understanding of 4 Vs of big data (volume, variety, velocity and veracity) as well as knowledge of how it is aggregated, analyzed and personalized in the context of decision making.^10^ They also need to be familiar with principles behind AI in general and for specific AI based tools in addition to learning to communicate with and through AI systems, communicating with patients whose health decisions will be impacted by AI and benefits and biases of AI applications during medical schools.^8^

Various advantages of use of AI in learning is provision of immediate feedback, enhancement of problem based learning with learning guided theory, identifying and responding to gaps in students’ knowledge, reduced need for teacher supervision, less costs and no potential harm to patients. Majority of virtual reality simulation program use an intelligent tutoring system (example TOUCH Project, ECHOCOM).^11^,^12^ AI use is mostly noticed in Undergraduate medical education perhaps due to presence of structured curriculum, which is generally lacking for Continuous medical education. Limitation for AI use for learning include necessity for structured curriculum to act as knowledge base for AI base program as well as concerns regarding quality of feedback as it requires system equipped with expert domain knowledge for contextually driven education.

AI is also used in assessment of learners like in assignment grading, automated essay scoring using clinical decision-making questions, evaluation of basic laparoscopic skills, grading of student case summaries, attendance tracking to name a few.^13^-^15^ Main barrier for AI limited use in assessments is lack of digitalization which impact on meeting the data pool requirements to develop AI based system. This limitation in much more pronounced in our educational institutions where we are using mostly non-digital tools for different domains including teaching, assessment, curriculum and evaluation. Also summative and sensitive nature of medical professional’s assessments, security issues regarding communication and possibility of malfunction or improper coding of AI system leading to wrong results further limits the use of AI systems of assessment. However, assessment in medical education is augmented by use of adaptive and programmatic assessments. In adaptive assessments, difficulty of questions is tailored according to individual being assessed based on their replies to earlier questions while optimization of learning outcomes in line with curriculum is the goal of programmatic assessments.^16^,^17^

AI has been used less frequently for curriculum review in medical education despite evidence of advantage of artificial neural networks (ANN), and support vector machine (SVM) in establishing relationships between variables along with providing an overview of the effectiveness of curriculum and students’ satisfaction with the program.^18^

Despite the evidence that AI competence is inevitably going to be added to the skills required of medical graduate in time to come, various challenges in implementing Artificial intelligence in medical education are noted. Two of the main challenges are insufficient time in curricular hours to adjust new content areas, and technical difficulties in development of AI applications, which are not only accurate but are clinically relevant too. It needs input from experts in medicine and education who will need to closely work with data scientists to ensure that AI tools works to complement medical teacher role rather than replacing teachers as was stated by Arthur C Clarke that “Any teacher who can be replaced by a machine should be!”. 19 Data Security, issues of privacy and confidentiality of learners’ data also need to be considered on a priority basis to ensure AI acceptance in medical education by relevant stakeholders. If AI is introduced in early years in medical students’ curriculum, the need to balance teaching and learning, students receive from Healthcare professionals and machines is needed as there is no substitute for human touch in medicine. Teaching soft skills in medicine (appreciation of ethics, communication skills, working in an empathic manner and leadership skills) is likely to increase in importance with introduction of AI in medical education.^8^

Medical schools currently lack the faculty expertise required to teach AI in medicine content. Lack of expertise in teaching AI to medical students can be addressed by increasing interfaculty collaborations between healthcare experts, engineering and computer sciences faculties. Use of AI tools to teach skills like ECG, Echo along with overview of how algorithm are developed, appreciation of broader principles of role of AI in decision making, predicting probabilities can be incorporated in medical school curriculum. To increase the awareness as well as highlight the importance of AI in medical education, authors have designed a six months’ course “Certificate in Online and Distance Education” (CODE) in which AI in relation to learning management system (LMS) given special consideration. Participants of the course will be learning these AI based LMS and how it will be helpful for student learning. 20

To conclude, with advancements in technology, role of AI in medical education is going to increase and new roles for medical students and doctors will be opened in future based on competency in using AI. Medical education needs to move beyond traditional biomedical and clinical sciences focus and engage with technology enhanced learning and AI. It is time for medical institutions to consider curricular reforms, including content related to AI and Machine learning as part of their curriculum, alongside emphasis on empathy and integrity. It will ensure that their graduates are ready to embrace these AI tools and are prepared to work in healthcare environment transformed by Artificial intelligence. The opportunities of use of AI in medical education is vast but we in Pakistan needs to start sensitizing our faculty and students now by taking small steps as pointed out by Turing in 1950, “We can only see a short distance ahead, but we can see plenty there that needs to be done”.^21^
